# Celebrating 30 years of partnership with the European field epidemiology training programmes

**DOI:** 10.2807/1560-7917.ES.2026.31.35.202609031

**Published:** 2026-09-03

**Authors:** Eurosurveillance editorial team

**Affiliations:** 1 European Centre for Disease Prevention and Control (ECDC), Stockholm, Sweden

**Keywords:** public health, Europe, field epidemiology training programmes, EPIET

For decades, *Eurosurveillance* and the European field epidemiology training programmes (FETPs) have had a long-standing collaboration. Back in the mid-1990s, the European Programme for Intervention Epidemiology Training (EPIET) and *Eurosurveillance* were created as part of a larger European public health vision with a similar philosophy. Developing and strengthening of dedicated disease-specific networks, including networks in training and communications, were considered essential for the evolving European communicable disease surveillance capacity [1,2]. Already in 1996, the January issue of *Eurosurveillance* featured an article on an *Outbreak of Escherichia coli O157 in Sweden* involving an EPIET fellow, and the April issue presented an update of developments of the training programme [3,4].

Thirty years later, the upcoming World Field Epidemiology Day, observed on 7 September 2026, is a worthwhile occasion to reflect on the joint contributions to field epidemiology of these successful initiatives. Both *Eurosurveillance* and EPIET were integrated into the European Centre for Disease Prevention and Control (ECDC) in 2007, where EPIET is now part of the ECDC Fellowship Programme together with the European Public Health Microbiology programme (EUPHEM, established in 2008) and they have since continued to collaborate. *Eurosurveillance* has two article categories dedicated to outbreak detection, investigation and control: Rapid communications and Outbreak articles. Reflecting their specific assignments in national and multi-country outbreak investigations, fellows have frequently contributed articles in these categories, including important rapid communications with cross-border relevance, often in cooperation with their peers in Europe. A recent example is a communication by van den Berg et al. on the multi-country investigation and response of an Andes virus outbreak linked to cruise ship travel [5].

In this issue, Brinkwirth et al. report a mixed-methods evaluation of Germany’s 30-year Postgraduate Training for Applied Epidemiology (PAE) programme, an EPIET-associated programme since the beginning. The authors found the programme was instrumental in developing and retaining a skilled public health workforce, with most of the 126 graduates remaining in German public health, contributing to outbreak response and leadership, and strengthening collaboration through shared expertise and professional networks [6]. While the evaluation results were overall encouraging, authors acknowledge that expanding the reach of FETPs *across all levels of the public health system, particularly at regional and local levels, could further strengthen capacity.* Of note, Germany’s PAE fellows have contributed 37 peer-reviewed publications to *Eurosurveillance* between January 1997 and February 2026, from the successful and impactful programme.

*Eurosurveillance* editors have also contributed over many years to building capacity in scientific writing and publishing through workshops and seminars during training modules of the ECDC Fellowship programme (EPIET and EUPHEM) and the EPIET-associated programmes. These activities have brought the journal editors closer to an important group of contributors and vice versa. On this basis, long-term partnerships have been formed and strengthened. Over time, many EPIET alumni have been nominated as editorial advisors, representing their countries on the *Eurosurveillance* editorial board or have joined the board as associate editors in their personal professional capacity.

This year, a new area of collaboration with the FETPs focusses on training and capacity-building of critical appraisal skills and providing written feedback through peer reviewing of scientific articles. In late 2022, *Eurosurveillance* launched a pilot initiative, welcoming senior reviewers to invite early-career researchers (ECR) to conduct a joint peer review. This pilot was designed not only to facilitate opportunities for mentorship and capacity-building, but also to diversify the journal’s reviewer pool and ensure that ECRs receive proper recognition for reviewing. To date, ca 80 pairs have conducted a co-review for the journal. Given the positive reception and encouraging feedback for the initiative, we have joined forces with FETPs to systematically engage the fellows in co-reviewing of *Eurosurveillance* articles. Following a kick-off event in Cambridge with the UK’s FETP in mid-April, a co-review collaboration was officially launched in June and is now underway with great enthusiasm from both sides. A similar collaboration was launched last week with the ECDC Fellowship Programme (EPIET and EUPHEM) and its associated fellowship programmes to invite fellows to perform a co-review with their supervisor on a voluntary basis.

On the occasion of our 30^th^ anniversary, we wish all our partners in the European FETPs a Happy Birthday and we look forward to continuing our creative and fruitful 30 years of collaboration as part of a strong network for public health over at least the next 30 years!
